# Psychometric properties and clinical usability of the cognition in daily life scale in patients with acquired brain injury in clinical care settings

**DOI:** 10.1177/02692155251372114

**Published:** 2025-09-23

**Authors:** Anne-Fleur Domensino, Johanna MA Visser-Meily, Jacoba M. Spikman, Caroline van Heugten, The CDL Study Group

**Affiliations:** 1 School for Mental Health and Neuroscience (MHeNS), 168089 Maastricht University, Maastricht, The Netherlands; 2 Limburg Brain Injury Centre, Maastricht, The Netherlands; 3 Department of Rehabilitation, Physical Therapy Science and Sports, UMC Utrecht Brain Center, 526115 University Medical Center Utrecht, Utrecht, The Netherlands; 4 Center of Excellence for Rehabilitation Medicine, Brain Center, University Medical Center Utrecht and De Hoogstraat Rehabilitation, Utrecht, The Netherlands; 5 Division of Neuropsychology, Department of Neurology, 10173 University Medical Center Groningen, Groningen, The Netherlands; 6 Department of Neuropsychology and Psychopharmacology, 396107 Faculty of Psychology and Neuroscience, Maastricht University, Maastricht, The Netherlands

**Keywords:** Cognitive functioning, daily life, brain injury, observation, measurement instrument

## Abstract

**Objective:**

Evaluate the internal consistency, inter-rater and test–retest reliability, convergent and divergent validity and clinical usability of the Cognition in Daily Life scale for patients with acquired brain injury.

**Design:**

Validation study.

**Participants:**

A total of 75 patients with acquired brain injury (mostly male [*n* = 47, 68%]; mean age 67 years) were recruited from inpatient care facilities. Sixty participants (81%) had sustained a stroke.

**Main measures:**

*Outcome measure:* Cognition in Daily Life scale. *Reference measures:* Utrecht Scale for Rehabilitation-Cognition subscale, Montreal Cognitive Assessment, Barthel Index, Hospital Anxiety and Depression Scale and Fatigue Severity Scale.

**Results:**

After removing redundant items, all subscales of the Cognition in Daily Life scale demonstrated satisfactory internal consistency. Test–retest reliability was good (intraclass correlation coefficient [ICC] = 0.847), and inter-rater reliability was moderate (ICC = 0.615). Convergent validity was confirmed through moderately strong correlations between most subscales of the Cognition in Daily Life Scale and other measures of cognition. Cognition in Daily Life subscales generally did not correlate with the Hospital Anxiety and Depression Scale and Fatigue Severity Scale, indicating divergent validity. Moderate correlations with the Barthel Index suggested related, but distinct constructs. Clinicians found the Cognition in Daily Life scale easy to administer and relevant for practice, though time-consuming. They suggested layout improvements for greater usability.

**Conclusion:**

The Cognition in Daily Life scale is adequately valid, reliable and clinically usable for assessing cognition in daily life in patients with acquired brain injury in a clinical setting. Future research needs to evaluate the scale's sensitivity to change and its performance in other settings and populations.

## Introduction

Many patients who sustained acquired brain injury encounter cognitive impairments. The impact of these cognitive impairments on everyday functioning is referred to as *cognition in daily life*. Cognition in daily life entails observable behaviour, which is influenced by cognitive impairment, personal styles and beliefs and environmental factors. It involves cognitive processes such as attention, memory and planning that underlie everyday activities. For example, cooking a meal requires remembering ingredients, focusing attention and organizing steps while dealing with daily distractors. Impaired cognition in daily life can lead to limitations in activities such as mobility and self-care,^1^ and restrict individuals from participating in society when they want to return to work^2^ or household activities.^3^ Consequently, it is important to address cognition in daily life at an early stage after the injury.

Currently, cognitive impairments are effectively assessed with standardized cognitive tests in controlled settings, such as a quiet testing room. These tests allow for measuring neurocognitive impairment without the influence of environmental distractors. However, the step from identifying separate cognitive impairments in a structured way to interpreting how these different impairments result in limitations in daily life is a difficult one.^4^ Discrepancies between test results and daily functioning are not uncommon: on the one hand, standardized tests often pose fewer demands than the complex situations individuals face in daily life. On the other hand, individuals may adequately compensate for their cognitive impairments in daily life by using strategies and environmental adaptations, which cannot be applied during standardized testing. Since the aim of rehabilitation is to optimize activities and participation, assessments with high ecological validity are needed. Measures that allow identification of the effect of single or combined cognitive impairments in situations in ‘the real world’ can fulfil this need.

Measuring the extent to which patients can use cognitive functions in daily life and compensate for any cognitive impairment can contribute to clinical diagnosis, treatment design and discharge planning. We put forward that a standardized method is not yet available for this purpose. Currently, cognition in daily life is often assessed in an unstructured manner, such as by taking observational notes in patient files^5,6^ which can pose challenges for interpretability and reliability. Alternatively, structured assessments focused on specific assigned tasks, such as the Allen Cognitive Level Screen,^7^ may not accurately reflect cognitive functioning in real-life situations. Additionally, many structured observation instruments assess task performance instead of cognition in daily life, such as the Assessment of Motor and Process Skills.^8^ Moreover, more generic rehabilitation outcome measures, such as the Utrecht Scale for the Evaluation of Rehabilitation,^9^ include only a few items related to cognition and thus provide limited insight into the complexity of cognitive functioning in daily life. A structured observation of cognition in daily life, however, can provide information on the level of real-life cognitive functioning after acquired brain injury, making it a valuable addition to neuropsychological assessment and enabling the tracking of changes over time (e.g., when tracking improvement in daily functioning during rehabilitation, or to evaluate changes when patients return to work). This way, clinicians are assisted in unravelling the complexity of acquired brain injury consequences, distinguishing physical from cognitive problems, and encouraged to speak a common language when discussing cognition in the context of (rehabilitation) treatment. As a comprehensive and standardized observation scale for cognition in daily life was until recently unavailable, we developed the *Cognition in Daily Life scale.*

The Cognition in Daily Life scale was designed to facilitate structured observation and interpretation of behaviour in a variety of settings, with particular relevance for multidisciplinary (rehabilitation) environments. By adding scoring to the observed behaviours, assessment of progress and changes over time is enabled. This is important because outcome assessment on the level of activities and participation is recommended as part of neuropsychological treatment.^10^ Its measurement aim aligns well with the biopsychosocial aims of rehabilitation^11^ as it supports assessment of how individuals with brain injuries manage cognitive challenges in daily activities, social roles and their environment. By evaluating cognitive functioning within the context of daily life, the Cognition in Daily Life scale provides insight into the extent to which patients can effectively employ compensatory strategies, such as those learned during cognitive rehabilitation^12^ to manage cognitive impairments. This highlights the potential value of the Cognition in Daily Life scale as a tool for professionals from various disciplines working with individuals with acquired brain injury in rehabilitation settings. The instrument was developed through an iterative process of item generation, followed by expert evaluation to ensure content validity.^13^ Before implementing the measure, it is essential to assess the reliability, construct validity and clinical usability of the instrument to ensure its efficacy, validity and reliability in practice.

Therefore, the purpose of the current study was to further evaluate the psychometric properties (internal consistency, inter-rater and test–retest reliability and construct validity) as well as the clinical usability of the Cognition in Daily Life scale in inpatient care for patients with acquired brain injury.

## Methods

### Design and participants

This mixed-methods observational study recruited participants with acquired brain injury from 17 healthcare institutions across the Netherlands. Nine institutions were facilities for specialized medical rehabilitation, five concerned facilities for geriatric rehabilitation services, two institutions provided assisted living and one was a mental health care institution for patients with acquired brain injury and psychiatric problems. A convenience sampling strategy was employed, wherein a designated clinician at each institution was instructed to include a minimum of five patients from their respective populations. Eligible participants had to be over 18 years of age, have sustained any type of medically confirmed acquired brain injury (i.e., stroke, moderate-severe traumatic brain injury, hypoxia, intoxication, infection or brain tumour), be admitted to or living at the healthcare institution, be expected to stay at the facility for at least two more weeks and be able to perform basic daily activities (not being bedridden). Participants were excluded from the study if they were diagnosed with a neurodegenerative disease (i.e., Alzheimer's disease, Parkinson's disease or multiple sclerosis) or were unable to provide informed consent. There were no restrictions regarding the time since admission or time since injury for the participants.

Data were collected between July 2023 and March 2024. This study was approved by the Medical Ethical Committee of [anonymized] (reference number METC 2023-3693) and by the local committees of the healthcare institutions, where necessary. All participants provided written informed consent.

### Demographic and injury-related characteristics

Demographic characteristics of participating patients (age, gender and living situation) were assessed using a self-report questionnaire. The designated clinician in each institution retrieved information about the patients’ acquired brain injury (type of injury, time since injury, and presence of previous brain injury) from their medical files.

### The Cognition in Daily Life scale

The Cognition in Daily Life scale is a recently developed instrument for the assessment of cognition in daily life. The Dutch draft version of the Cognition in Daily Life scale, which was used in the current study, comprises 65 items rated on severity and frequency across six domains: alertness, processing speed & attention (12 items), perception (7 items), orientation & memory (11 items), actions (3 items), language & communication (16 items) and task behaviour (16 items). The relevance of the selected items and the expected usability and face validity of the scale were previously evaluated positively by an international panel of experts in measuring cognition.^13^ The Cognition in Daily Life scale can be administered by any clinician who is involved in the daily care for the patient, is specialized in working with individuals with brain injuries, and has experience in observing behaviour. The scale is completed through direct daily observations by a healthcare professional, based on informal interactions and without interfering with the patient's daily routine. Drawing from these observations, the healthcare professional rates the frequency and severity of each behaviour. As part of the manual, clinicians are instructed to score only behaviours that are likely caused by cognitive problems, and not those resulting from other issues such as visual, auditory or motor impairments. The estimated administration time of the Cognition in Daily Life scale is 20 min. A total score for each cognitive domain is then calculated by multiplying the frequency (0 – never to 3 – always) and severity (0 – no to 3 – severe) scores for each item, and summing the results. The scoring system of the Cognition in Daily Life scale was based on the neuropsychiatric inventory,^14^ which has been demonstrated to be valid and feasible for use by healthcare professionals.^15^ The subscale scores are calculated using the following formula: (sum of item scores per subscale) / (number of completed items) * 100. Each item may also be marked as ‘not observed’, indicating that the clinicians had not had the opportunity to observe this specific behaviour. Items marked as ‘not observed’ are excluded from the subscale score calculation.

### Reference measures

The Utrecht Scale for the Evaluation of Rehabilitation^9^ is a clinician-reported screener on the global outcomes of inpatient rehabilitation. The cognition subscale measures cognitive independence as perceived by a clinician, with scores ranging from 0 to 50, where higher scores indicate greater independence. It consists of 10 items across the domains of communication, cognition and behaviour (i.e., ‘*Retains information. This includes working memory, short-term and long-term memory*’). Each item is rated on a 6-point scale, with scores reflecting levels of independence ranging from no difficulty with or without assistance to full dependence on others. The cognitive subscale of the Utrecht Scale for the Evaluation of Rehabilitation is considered reliable and valid.^^9^^

The Montreal Cognitive Assessment^16^ is a one-page cognitive screening test used to assess attention, concentration, executive functions, memory, language, visuospatial skills, abstraction, calculation and orientation. It produces a compound score of cognitive functioning ranging from 0 to 30, with higher scores indicating better cognitive functioning. Scores below 26 indicate cognitive impairment.^16^ The Montreal Cognitive Assessment was found to be a valid and reliable instrument for assessing global cognitive impairment after acquired brain injury.^17^

Clinicians completed the Barthel Index^18^ to measure independence in activities of daily living. The scale consists of 10 items and scores range from 0 to 20, with higher scores indicating greater independence. The Barthel Index is a frequently used measurement instrument that was found to be valid and reliable for use in acquired brain injury populations.^19^

Symptoms of anxiety and depression were measured with the anxiety as well as the depression subscale of the Hospital Anxiety and Depression Scale.^20^ Both subscales range from 0 to 21, with higher scores indicating more symptoms. The Hospital Anxiety and Depression Scale is considered valid and reliable for use in acquired brain injury populations.^21^

The Fatigue Severity Scale^22^ was used to measure fatigue. It is a short 9-item self-report questionnaire for the assessment of the impact of fatigue and is rated on a 7-point scale. The first two items of the Fatigue Severity Scale were omitted for the current study, as previous research showed better performance of the 7-point scale in stroke population.^23^

### Feasibility interviews

To evaluate the clinical usability of the Cognition in Daily Life scale, the principal investigator (AD) conducted a semi-structured interview with one clinician from each participating institution who had used the Cognition in Daily Life scale in the study, after data collection was completed. In four of the participating institutions, two (instead of one) healthcare professionals volunteered to participate in the interview, resulting in a total sample of 21 interviewees. The interview guide (Supplementary Table A) was based on existing implementation frameworks for healthcare practice and contained questions on fidelity, adoption, appropriateness and feasibility.

### Procedure

Eligible patients were approached by clinicians (psychologists, occupational therapists, physiotherapists or nurses) from the participating institutions. These clinicians were responsible for administering the baseline measurements and completing the Cognition in Daily Life scale. Participating clinicians were instructed to thoroughly read the instrument manual and items of the scale before participant inclusion. They did not receive any specific training on how to use the Cognition in Daily Life scale.

Potential participants were provided with an information letter and the contact details of the principal investigator (AD). Upon agreeing to participate, patients signed the informed consent form at the baseline meeting (T0), during which the Montreal Cognitive Assessment, Barthel Index, Hospital Anxiety and Depression Scale and Fatigue Severity Scale assessments were completed. The observation period started the day after the baseline measurements and lasted for seven days. To determine the inter-rater reliability of the Cognition in Daily Life scale, two clinicians independently observed the patients as they went about their daily activities without offering any prompts or instructions (i.e., ‘*Can you show me how you would brush your teeth?*’). At the end of the observation period, both clinicians (observer 1 and observer 2) independently completed the Cognition in Daily Life scale and the Cognition subscale of the Utrecht Scale for the Evaluation of Rehabilitation (T1). To determine the test–retest reliability of the Cognition in Daily Life scale, one of these clinicians observed the patient again during the following seven days and completed the Cognition in Daily Life scale a second time (T2). A relatively short interval between test and retest was chosen to minimize score discrepancies unrelated to test–retest reliability, as patients were expected to improve due to their treatment. Clinicians were not specifically trained to use the Cognition in Daily Life scale but were instructed to thoroughly review the instrument before the initial observation period. After finishing data collection, feasibility interviews were scheduled based on the availability of the clinicians. These interviews, conducted via video calls or phone calls, had an approximate duration of 20 minutes.

### Data analyses

The score distribution of the items of the Cognition in Daily Life scale at T1 was evaluated in terms of mean, standard deviation, range, median and interquartile range, skewness and floor and ceiling effects. Floor and ceiling effects were evaluated for all subscales and were present if at least 15% of the participants obtained the highest or lowest score on a specific subscale.^24^ Internal consistency was evaluated based on the first of both Cognition in Daily Life scale measurements using Cronbach's alpha for each cognitive subscale. This approach aligns with our goal of measuring different behaviours that, while potentially influenced by different underlying causes, are grouped within a domain due to shared behavioural characteristics. The total scale was not analysed as a whole, as each domain reflects a distinct set of behaviours rather than a single underlying construct. Item-total correlations of at least 0.30 were considered satisfactory.^24^ Overall, Cronbach's alpha was considered acceptable if it was higher than 0.70.^24,25^ However, because the subscales have varying lengths, the α-values were interpreted in the context of each subscale's length.^26^ Items were removed from the subscales based on a combined evaluation of the corrected item-total correlations, scale reliability if the item was deleted, the total number of items in the subscale, and conceptual meaning. After removing redundant items, the internal consistency was recalculated, and further analyses were performed using only the retained items.

Inter-rater reliability was calculated using intraclass correlation coefficient (ICC) analyses in a two-way random model, as the raters differed for each participant due to the multicentre setup of the study. Intraclass correlation coefficients were calculated based on absolute agreement. Test-retest reliability was calculated using ICCs between the two sets of results taken at different time points and was based on a two-way mixed model since the same rater completed the measurement twice for each participant. Intraclass correlation coefficients of 0.50–0.75 indicated moderate reliability, 0.75–0.90 good reliability and >0.90 excellent reliability.^27^

Convergent validity, which refers to the extent to which the Cognition in Daily Life scale corresponds to other established measures of cognitive functioning, was evaluated using bivariate Spearman correlation analyses between all subscales of the Cognition in Daily Life scale and both the Montreal Cognitive Assessment and Cognition subscale of the Utrecht Scale for the Evaluation of Rehabilitation. Moderate to strong significant correlations were expected between the Cognition in Daily Life scale subscales and the Cognition subscale of the Utrecht Scale for the Evaluation of Rehabilitation. Given that the Montreal Cognitive Assessment assesses cognitive impairment within a controlled environment, it is anticipated to measure a related, but not identical construct to the Cognition in Daily Life scale and therefore result in weak to moderate significant correlations. Divergent validity, which assesses the degree to which the Cognition in Daily Life scale does not correlate with measures of different constructs, was calculated using bivariate Spearman correlation analyses between the Cognition in Daily Life scale and the Anxiety and the Depression subscale of the Hospital Anxiety and Depression Scale, Fatigue Severity Scale and Barthel Index. No correlations were expected between the subscales of the Cognition in Daily Life scale and the subscales of the Hospital Anxiety and Depression Scale or Fatigue Severity Scale, respectively. The Barthel Index measures activities of daily living, which are dependent on cognitive functioning levels. Therefore, the associations between the Barthel Index and the Cognition in Daily Life scale are expected to be significant but weak. Correlation coefficients were interpreted as weak (0.10–0.29), moderate (0.30–0.49) or strong (≥0.50).^28^

Regarding qualitative data analysis, summary transcriptions were taken from the usability interviews. The data were analysed using deductive framework analysis,^29^ in which transcription data were categorized based on the predefined usability aspects outlined in the interview guide by the principal investigator (AD).

Sample size recommendations for psychometric studies vary by the type of analysis and scale characteristics. For internal consistency, subscales with a moderate number of items (around 7–15) generally require between 50 and 100 participants to yield reliable estimates of Cronbach's alpha.^30^ Reliability analyses including inter-rater and test–retest reliability, typically require a minimum of 50 participants to provide robust ICC estimates.^31^ For construct validity, sample sizes between 29 and 85 participants are required to detect moderate correlations (*r* = 0.3–0.5) with 80% power and an alpha of 0.05. The current study, with a final study sample of 75, is therefore sufficiently powered for most of the analyses.

## Results

Seventy-five patients of the 17 healthcare institutions agreed to participate. The mean age of the sample was 66.7 years, most participants were men (*n* = 47, 68%), who were most frequently admitted to a rehabilitation centre (*n* = 44, 68%). The most common cause of injury was a stroke (*n* = 60, 81%). There was large heterogeneity in terms of time since injury (range 0–458 months), resulting from the variety in sampling settings (acute to long-term care). On average, participants scored below the cut-off for normal cognitive functioning on the Montreal Cognitive Assessment (M = 20.8 (4.7); n = 54 (87%)). A full description of the sample is detailed in Table 1.

**Table 1.** Descriptives of demographic characteristics, injury type and duration and baseline scores on reference measures for the study sample (*n* = 75).

Variable	M; range (SD) / *N* (%)
Age	66.7; 18–96 (15.7)
Gender (male)	47 (68%)
Type of institution	
Rehabilitation care	44 (60%)
Geriatric rehabilitation care	16 (22%)
Assisted living	8 (11%)
Mental health care	5 (7%)
Type of injury	
CVA	60 (81%)
TBI	8 (11%)
Other (hypoxia, intoxication, infection, tumour)	6 (8%)
Time since injury in months	26.2; 0–458 (15.9)
ADL independence (BI)	16.1; 1–20 (5.0)
Cognitive functioning (MoCA)	20.8; 8–30 (4.7)
*Proportion below cut-off*	54 (87%)
Fatigue (FSS)	4.1; 1–6.9 (1.5)
Anxiety (HADS-A)	5.13; 0–15 (3.6)
Depression (HADS-D)	4.9; 0–17 (3.9)

CVA: Cerebrovascular accident; TBI: Traumatic Brain Injury; BI: Barthel Index; MoCA: Montreal Cognitive Assessment; FSS: Fatigue Severity Scale; HADS-A: Hospital Anxiety and Depression Scale – Anxiety Subscale; HADS-D: Hospital Anxiety and Depression Scale – Depression Subscale.

Scores on the Cognition in Daily Life scale at T1 for observer 1 were available for all participants (*n* = 75). Scores at T1 for observer 2 were missing for ten participants (*n* = 65), and scores on the Cognition in Daily Life scale at T2 were missing for four participants (*n* = 71). Median scores were highest for the *alertness, processing speed and attention* subscale, meaning that most problems were observed in this cognitive domain. Median scaled subscale scores ranged from 0.96 to 11.11 and were right-skewed for all subscales, indicating that patients were more likely to obtain scores below average than above. Floor effects were present in all subscales, indicating that a proportion of patients did not show any problems in one or more subdomains. No ceiling effects were present. All descriptive statistics of the Cognition in Daily Life scale at T1 are displayed in Table 2. Scoring distributions of all subscales are displayed in Supplemental Figure A.

**Table 2.** Unscaled subscale scores as provided by observer 1 at T1.

Subscale	Median (IQR)	Skewness	Floor (%)
Alertness, processing speed & attention	11.11 (3.8–25.0)	0.734	8 (10.7)
Perception	0.96 (0.0–3.5)	2.675	43 (57.3)
Orientation & memory	2.92 (0.6–10.3)	1.626	18 (24.0)
Actions	1.03 (0.0–3.1)	5.189	56 (75.5)
Language & communication	2.36 (0.3–8.9)	2.235	24 (32.0)
Task behaviour	6.83 (2.6–16.4)	1.907	9 (12.0)

### Internal consistency

Internal consistency of the Cognition in Daily Life scale was acceptable for all but one subscale (Actions; α = .605). The *task behaviour* subscale had exceptionally high internal consistency (α = .948). A full overview of the internal consistency of each subscale, as well as all corrected item-total correlations and Cronbach's α for the subscales when each item would be removed from its corresponding subscale is included in Supplemental Table B and C. Seven items showed a corrected item-total correlation of <.30. Five of these were removed (1, 16, 33, 47 and 49), one item (23) was merged with a similar item (24). One item (14) was retained because of theoretical relevance, as it describes prosopagnosia, which is a rare but impactful condition. Five other items (item 27, 36, 52, 57 and 59) were removed despite having satisfactory corrected item-total correlations, as they reflected behaviours that were either too covert for clinicians to observe or not applicable to an inpatient environment. This resulted in low completion rates. Another four items (18, 22, 58 and 61) were rephrased based on feedback from clinicians and/or response patterns. Further analyses were run without the excluded items. Internal consistency of the final subscales is displayed in Table 3. The final version of the questionnaire, consisting of 51 items, is displayed in Table 4.

**Table 3.** Internal consistency of final cognition in daily life scale subscales.

Subscale	Final number of items	Valid N	Cronbach's α
Alertness, processing speed & attention	11	68	.837
Perception	6	44	.722
Orientation & memory	9	58	.781
Actions	2	71	.801
Language & communication	10	48	.836
Task behaviour	13	57	.934

*Note*. Participants with missing values (including ‘not observed’) on individual items were excluded from subscale analyses.

**Table 4.** Items of the final cognition in daily life scale.

Item	Description
*Awareness, processing speed, & attention*	
1	The patient is not alert during activities (misses relevant information or events).
2	The patient cannot keep up with the pace in which information is presented (e.g., reading subtitles or following a conversation) and thus misses information.
3	The patient performs tasks slowly (e.g., getting dressed slowly when under pressure to leave the house).
4	The patient responds slowly (e.g., in a conversation or to instructions).
5	The patient's attention is not automatically drawn to relevant information from the environment (e.g., does not notice a person who just walked into the room).
6	The patient pays insufficient attention to one side of his/her body or one side of space (i.e., neglect) (e.g., forgets to wash the affected side, bumps into objects on the affected side).
7	The patient cannot stay focused during an activity for an extended period of time (e.g., when following a conversation or performing a task).
8	The patient is not able to focus on a task when there are distractions.
9	The patient has difficulty doing two things at the same time (e.g., talking and walking).
10	The patient has difficulty switching between activities or sources of information.
11	The patient cannot stay focused on an activity for an extended time (e.g., when following a conversation or performing a task).
*Perception*	
12	The patient does not recognise objects or mistakes them for something else.
13	The patient does not recognise familiar faces.
14	The patient does not recognise letters, symbols, and/or numbers.
15	The patient gets lost in a familiar environment.
16	The patient gets lost in an unfamiliar environment (e.g., another department in the institution or location outside the institution).
17	The patient cannot estimate the distance to or position of an object in relation to him/herself and therefore misses the object when reaching for it.
*Orientation & memory*	
18	The patient does not know where he/she is.
19	The patient does not know if it is morning, afternoon or evening.
20	The patient does not know what day, month or year it is.
21	The patient does not know his/her own personal data or important personal events from the past.
22	The patient does not show up to scheduled appointments.
23	The patient cannot recall recent information that has to be applied immediately (e.g., instructions on how to perform a task).
24	The patient is not able to continue an activity after an interruption (e.g., when asked a question), because the patient does not remember what he/she was doing.
25	The patient has difficulty remembering recent events, not even when asked to recall a specific event (e.g., remember what we had for dinner yesterday?).
26	The patient doesn’t spontaneously remember previously retained information but does recognize it when it's mentioned (e.g., remember we had pasta for dinner yesterday?)
*Actions*	
27	The patient performs the parts of an action in an incorrect order (i.e., apraxia) (e.g., when getting dressed).
28	The patient uses objects in an incorrect, clumsy or unsafe way.
*Language & communication*	
29	The patient does not understand spoken language.
30	The patient cannot name the correct words or names.
31	The patient says other words than what he/she means (paraphasia).
32	The patient cannot verbally express what he/she means (exactly).
33	The patient cannot formulate grammatically correct sentences.
34	The patient cannot understand written text.
35	The patient cannot write.
36	When telling a story, the patient cannot distinguish between what is important and what is less important (is not to the point).
37	The patient skips from one subject to another (i.e., tangential speech)
38	The patient interrupts others in a conversation.
*Task behaviour*	
39	The patient cannot really determine what needs to be done to achieve his/her goal when performing a new activity.
40	The patient sets unrealistic goals (e.g., being able to work full-time immediately after being away for a long time).
41	The patient cannot think of solutions to a problem.
42	The patient does not prioritise his/her activities.
43	The patient does not apply a step-by-step or efficient approach during an everyday task (e.g., when looking for an object).
44	The patient is not able to execute a scheduled plan.
45	The patient does not perform everyday activities on his/her own initiative but performs these actions readily with prompting (e.g., daily routines such as walking the dog or washing the dishes after dinner).
46	The patient starts an activity without thinking.
47	The patient cannot control him/herself (e.g., eats food when in sight of it or does not refrain from making inappropriate remarks).
48	The patient is upset when plans change.
49	The patient cannot switch to new behaviour/another procedure if the situation requires this.
50	The patient does not monitor what he/she is doing and fails to make corrections if necessary.
51	The patient fills the gaps in his/her memory or perception with incorrect information and is not able to judge the representation as false (confabulates).

*Note:* The original items were in Dutch and were translated into English by a medical translator. Items were partly based on the Handbook of Interdisciplinary Cognitive Treatment^32^ and were used with permission.

### Reliability

Inter-rater reliability of the Cognition in Daily Life scale was moderate (ICC = .615) and increased with combined ratings (ICC = .761). Test–retest reliability of the Cognition in Daily Life scale was good (ICC = .847). Paired-samples Mann–Whitney *U* tests showed that summed item scores were significantly lower over time in patients who received medical rehabilitation care (Mdn T1 = 27; Mdn T2 = 16; *P* = 0.014).

### Construct validity

All but two subscales (*actions and tasks*) showed weak to moderate, significant correlations with the Montreal Cognitive Assessment. All but one subscale (*actions*) showed moderate to strong correlations with the Cognition subscale of the Utrecht Scale for the Evaluation of Rehabilitation, confirming convergent validity. Only the *memory* subscale showed a weak but significant negative correlation with the Fatigue Severity Scale. None of the Cognition in Daily Life scale subscales correlated with the Hospital Anxiety and Depression Scale, indicating divergent validity. A moderate correlation between the *attention* and *tasks* subscales and the Barthel Index indicated related, but distinct constructs. A full overview of the correlations between Cognition in Daily Life scale subscales and reference measures is displayed in Table 5. The relationships between the subscales of the Cognition in Daily Life scale and both reference measures for construct validity are visualised in Supplemental Figure B.

**Table 5.** Correlations between reference measures and cognition in daily life scale subscales.

	Alertness, processing speed & attention	Perception	Orientation & memory	Actions	Language & communication	Task behaviour
MoCA	−.32*	−.26*	−.27*	−0.23	−.37**	−0.07
USER-C	−.53**	−.54**	−.41**	−0.26	−.40**	−.63**
BI	−.38**	−.09	−.17	−0.21	−.11	−.33**
FSS	−.14	−.04	−.25*	−0.23	−.08	−.15
HADS-A	.04	−.12	−.05	−0.10	−.05	−.04
HADS-D	−.12	.12	−.04	−0.12	−.22	−.01

MoCA: Montreal Cognitive Assessment; USER-C: Utrecht Scale for Evaluation of Rehabilitation, Cognition Subscale; BI: Barthel Index; FSS: Fatigue Severity Scale; HADS-A: Hospital Anxiety and Depression Scale, Anxiety Subscale; HADS-D: Hospital Anxiety and Depression Scale, Depression Subscale.

*Correlation is significant at the 0.05 level (two tailed).

**Correlation is significant at the 0.01 level (two tailed).

### Clinical usability

Semi-structured interviews (*n* = 21) were conducted with clinicians of each of the participating institutions. Most of these were female (*n* = 20, 95%). Nine (43%) were occupational therapists, five (24%) were psychologists, four (19%) were nurses, and three (14%) were physiotherapists.

Regarding fidelity, clinicians completed the Cognition in Daily Life scale according to the instructions, partially because it was administered as part of a research project instead of regular clinical practice. Some mentioned that they were unsure how to interpret the ‘severity’ scale and wondered whether they needed to score the severity of the problem from the perspective of the client or themselves. These clinicians remarked that some of their patients have limited awareness of their deficits, which is why they may not perceive their problems as severe. Although the clinicians expressed uncertainty about how to complete the questionnaire in such cases, all but one responded to the severity scale from their own perspective, as intended.

When looking at the domain of adoption, clinicians expressed their enthusiasm about the Cognition in Daily Life scale and were open to using it in their daily practice. Most expressed their intention to incorporate the Cognition in Daily Life scale into daily practice once it becomes available, although they would mainly use it for patients where specific questions about cognitive problems in daily life arise. The Cognition in Daily Life scale was of added value to the instruments for measuring cognition that are currently available, although some occupational therapists preferred their own observation techniques over the Cognition in Daily Life scale. All clinicians mentioned the long administration time of the Cognition in Daily Life scale; however, only some of them considered this an implementation burden. These clinicians would prefer the use of a short form or screening version of the Cognition in Daily Life scale.

Considering appropriateness of the instrument, clinicians all found the items of the Cognition in Daily Life scale relevant to their patient populations, and they appreciated the possibility to use the Cognition in Daily Life scale among patients with aphasia. One clinician remarked that she found it difficult to distinguish physical problems from cognitive ones when administering the Cognition in Daily Life scale. Clinicians noted that the Cognition in Daily Life scale helped to structure their observations and has added value for diagnostics and treatment planning in multidisciplinary teams.

In terms of feasibility, clinicians found the Cognition in Daily Life scale to be clear and easy to administer and did not consider formal training necessary prior to its use. They did mention that the total scores of the Cognition in Daily Life scale are difficult to interpret. Since no scaled scores were calculated for the draft version of the Cognition in Daily Life scale that was used as part of the study, clinicians were unable to compare subdomain scores.

Many clinicians remarked that the layout of the Cognition in Daily Life scale could be improved. They stressed the need for repeating the response options at the top of each page of the instrument to avoid administration errors. Moreover, most clinicians expressed a desire for a digital administration and scoring system for the Cognition in Daily Life scale, which would significantly increase the ease of administration and, consequently, implementation in clinical practice.

## Discussion

The current study evaluated the internal consistency, inter-rater reliability, test–retest reliability, convergent and divergent validity and clinical usability of the Cognition in Daily Life scale in an inpatient acquired brain injury population. The final version of the Cognition in Daily Life scale consists of 51 items across six subscales. The results show it is a valid and reliable tool for measuring cognition in daily life in this setting. Moreover, clinicians deemed the Cognition in Daily Life scale appropriate and feasible for use in this population and gave recommendations for further refinement of the instrument.

The inter-rater reliability of the Cognition in Daily Life scale was satisfactory and increased when ratings of two observers were combined. Similar levels of inter-rater reliability were found for observation scales of other overt behaviours, such as aggression.^33^ Given that observation scales are inherently prone to bias,^34^ their reliability tends to increase when multiple raters’ assessments are combined.^35^ Moreover, since distinguishing cognitive from physical problems can be challenging, involving multiple clinicians in the assessment process is recommended.

Post-hoc analysis of test–retest reliability indicated that scores significantly improved for participants in medical rehabilitation care only. Generally, inpatient medical rehabilitation care is aimed at a relatively young patient population and takes place in the subacute phase after the brain injury. Consequently, patients in this group were expected to show rapid improvement,^36^ and the observed changes for this group may represent an initial indication of the scale's responsiveness to change.

Regarding the construct validity of the Cognition in Daily Life scale, most of our a priori hypotheses were confirmed. The lack of strong correlations between the Montreal Cognitive Assessment and Cognition in Daily Life scale likely reflects their different aims; the Montreal Cognitive Assessment screens for cognitive *impairment*, whereas the Cognition in Daily Life scale measures cognitive *problems in daily life*. Some patients may perform well on cognitive screening but still struggle with daily functioning due to subtle impairments not captured by such tests.^37^ Conversely, some patients who showed cognitive impairment may be able to compensate for those using adequate strategies as a result of cognitive rehabilitation.

The lack of correlation between the *Actions* subscale of the Cognition in Daily Life scale and the Cognition subscale of the Utrecht Scale for the Evaluation of Rehabilitation can be explained by the lack of items representing apraxia-related problems in the latter. However, the finding that the *Actions* subscale did not significantly correlate with any of the reference measures is probably a consequence of the floor effect of this subscale, as it represents problems that are relatively rare or underdiagnosed.^38^

The Cognition in Daily Life scale showed promising results regarding clinical feasibility, although participating clinicians consistently remarked on the length of administration. We therefore removed and merged several items of the Cognition in Daily Life scale and resolved issues with the layout, resulting in greater ease of administration. Additionally, we improved the layout and the instructions for use according to the recommendations of the clinicians. Finally, a digital tool was developed to automate scoring and interpretation, aiming to standardize procedures and support integration into clinical workflows and patient records.

This study was conducted in a clinical rehabilitation setting, which may limit the direct generalizability of the findings to everyday, real-life contexts. However, the items of the Cognition in Daily Life scale are designed to reflect everyday cognitive challenges that extend beyond the clinical environment, making the scale relevant to real-world problems. While the scale has potential for use in outpatient contexts, further validation is required. Observations conducted outside of clinical environments, such as during home visits, may pose challenges to reliability. However, other observation scales have been proven to be useful in outpatient settings.^39^

The responsiveness of the Cognition in Daily Life scale needs to be further investigated. With confirmed responsiveness, the Cognition in Daily Life scale could be used to quantify the effects of cognitive rehabilitation, something that traditional tests of cognitive functioning are not suitable for.^40^ Furthermore, future research is necessary to establish clinical cut-off points that could guide referrals from the hospital or rehabilitation centre to independent or assisted living.

The current study is not without limitations. Our conclusions were based on a relatively small sample. However, post-hoc power analysis revealed that our sample yielded narrow confidence intervals (0.15–0.21) that do not exceed single reliability categories (e.g., ICC's of 0.75–0.9 indicating ‘*good reliability*’).^31^ Similarly, usability feedback predominantly originated from psychologists and occupational therapists with expertise in measuring cognition. Ease of administration among other disciplines may be less evident, and additional training may be required.

The Cognition in Daily Life scale consists of example problems across several cognitive domains, meaning that the default score for most individuals is around zero, with higher scores reflecting more cognitive difficulties. As a result, the score distribution is inherently right-skewed.^41^ Despite the fact that this has no implications for clinical practice, we were limited to the use of nonparametric tests for testing our hypotheses on convergent and divergent validity and changes over time. When evaluating the scale's responsiveness to change, floor effects need to be taken into account as they may limit the ability to detect improvement in individuals with low baseline scores.^24^

Lastly, we did not investigate all psychometric properties of the Cognition in Daily Life scale. Measurement error could not be evaluated because patients were not entirely stable over time,^24^ which is inherent to the population under study. Moreover, we did not study cross-cultural application of the Cognition in Daily Life scale, which is an important but often understudied feature of behavioural instruments for this population.^42^ In addition, we deliberately chose not to perform a factor analysis, as the scale's subdomains are intended as practical groupings rather than latent constructs. Cognitive domains in daily life are still poorly defined, and the scale's structure aims to offer a broad overview of relevant task behaviours, without implying strict separation between underlying cognitive impairments.

In conclusion, the final 51-item Cognition in Daily Life scale is a valid and reliable instrument for structured observation of cognition in daily life among brain injury patients in a clinical setting. The instrument shows implementation readiness in clinical care for these patients. It can be used to capture the impact of cognitive impairments on daily life, and changes therein during interventions aimed at compensating for these impairments. The use of the Cognition in Daily Life scale in multidisciplinary settings can enhance collaboration to understand complex behaviour. Integration of the Cognition in Daily Life scale with other results of a neuropsychological assessment can help neuropsychologists to translate the results of cognitive tests in a controlled environment to realistic real-life situations. Future research needs to be aimed at evaluating the validity of the Cognition in Daily Life scale in different populations and settings.

**Clinical messages**

- The Cognition in Daily Life scale is a valid and reliable observation scale for cognition in daily life (the cognitive processes underlying everyday activities) for inpatients with acquired brain injury.
- Its usability for clinicians from various disciplines ensures broad applicability.
- It raises awareness on cognitive problems in daily life and promotes shared language, facilitating coordinated care.

## Supplemental Material

sj-docx-1-cre-10.1177_02692155251372114 - Supplemental material for Psychometric properties and clinical usability of the cognition in daily life scale in patients with acquired brain injury in clinical care settings Supplemental material, sj-docx-1-cre-10.1177_02692155251372114 for Psychometric properties and clinical usability of the cognition in daily life scale in patients with acquired brain injury in clinical care settings by Anne-Fleur Domensino, Johanna MA Visser-Meily, Jacoba M. Spikman, Caroline van Heugten and The CDL Study Group in Clinical Rehabilitation
