# Supplementary material for: Psychometric properties and clinical usability of the cognition in daily life scale in patients with acquired brain injury in clinical care settings
Source: Clin Rehabil. 2025 Sep 23;39(11):1452–66. doi: 10.1177/02692155251372114 (PMC13103883; doi:10.1177/02692155251372114)
Supplement: sj-docx-1-cre-10.1177_02692155251372114 - Supplemental material for Psychometric properties and clinical usability of the cognition in daily life scale in patients with acquired brain injury in clinical care settings [file sj-docx-1-cre-10.1177_02692155251372114.docx]

**Supplemental materials**

**Supplemental Table A.** Interview guide for semi-structured feasibility interviews with participating clinicians.

| **Construct** | **Questions** |
| --- | --- |
| Adoption | - In your opinion, what criteria should an instrument meet to be used in practice?   - *Possible prompt: How long can an instrument be? What should it measure?* |
| Appropriateness | - Do you think the CDL is suitable for investigating cognitive problems within your patient group? |
| Feasibility | - Did you find the instrument complicated? Why or why not?   - Possible prompt: Were the instructions easy to follow?   - Possible prompt: Were the situations easy to assess? - Do you think the CDL could be immediately integrated into your practice, or would changes need to be made first?   - Possible prompt: For example, do you think healthcare providers would need extra training to use the instrument effectively in practice?   - Possible prompt: Do you think there is enough staff to use the instrument in practice?   - Possible prompt: Do you think there is enough time to complete the instrument in practice? - If not, what changes could we make to make the CDL more useful for you as a healthcare provider? |
| Fidelity | - Did you follow the steps in the instructions exactly as described? If not, what obstacles did you encounter that prevented you from following the instructions?   - Possible prompt: Did you observe the patient for one week?   - Possible prompt: Did you read through all the situations before you started scoring?   - Possible prompt: Did you complete the list fully, or did you skip parts? - If not, what obstacles did you encounter that prevented you from following the instructions? |

**Supplemental Table B.** Internal consistency of original CDL subscales.

| **Subscale** | **Valid N** | **Cronbach’s α** |
| --- | --- | --- |
| Alertness, processing speed & attention | 66 | .832 |
| Perception | 44 | .702 |
| Orientation & memory | 55 | .809 |
| Actions | 71 | .605 |
| Language & communication | 43 | .834 |
| Task behaviour | 48 | .948 |

*Note*. Participants with missing values (including ‘not observed’) on individual items were excluded from subscale analyses.

**Supplemental Table C.** Cronbach’s α for the subscales, item-subscale correlations, and Cronbach’s α when item was to be excluded.

| **Item number** | **Item description** | **Score of 0 (n)** | **%** | **Not observed (n)** | **%** | **Missing (n)** | **%** | **Min** | **Max** | **Item-total correlation** | **Cronbach's alpha of subscale when item is deleted** |
| --- | --- | --- | --- | --- | --- | --- | --- | --- | --- | --- | --- |
|  | **Alertness, processing speed and attention** |  |  |  |  |  |  |  |  |  |  |
| 1 | The patient shows signs of drowsiness as the day progresses. | 58 | 77.3 | 3 | 4 | 0 | 0 | 0 | 9 | 0.24 | 0.835* |
| 2 | The patient is not alert during activities (misses relevant information or events). | 48 | 64 | 1 | 1.3 | 0 | 0 | 0 | 6 | 0.32 | 0.831 |
| 3 | The patient cannot keep up with the speed in which information is provided (e.g. reading subtitles or following a conversation) and thus misses information. | 27 | 36 | 2 | 2.7 | 1 | 1.3 | 0 | 9 | 0.60 | 0.810 |
| 4 | The patient performs tasks slowly (e.g. getting dressed slowly when under pressure to leave the house). | 37 | 49.3 | 1 | 1.3 | 0 | 0 | 0 | 6 | 0.50 | 0.819 |
| 5 | The patient responds slowly (e.g. in a conversation or to instructions). | 42 | 56 | 0 | 0 | 0 | 0 | 0 | 9 | 0.51 | 0.818 |
| 6 | The patient's attention is not automatically drawn to relevant information from the environment (e.g. does not notice a person who just walked into the room) | 57 | 76 | 0 | 0 | 0 | 0 | 0 | 9 | 0.44 | 0.824 |
| 7 | The patient pays insufficient attention to one side of his/her body or one side of space (i.e. neglect) (e.g. forgets to wash the affected side, bumps into objects on the affected side). | 51 | 68 | 2 | 2.7 | 0 | 0 | 0 | 9 | 0.47 | 0.823 |
| 8 | The patient is not able to focus on a task (when there are no distractions). | 46 | 61.3 | 0 | 0 | 0 | 0 | 0 | 6 | 0.50 | 0.822 |
| 9 | The patient is not able to focus on a task when there are distractions. | 22 | 29.3 | 1 | 1.3 | 0 | 0 | 0 | 9 | 0.59 | 0.811 |
| 10 | The patient has difficulties doing two things at the same time (e.g. talking and walking). | 17 | 22.7 | 0 | 0 | 0 | 0 | 0 | 9 | 0.63 | 0.807 |
| 11 | The patient has difficulties switching between two simultaneous activities or sources of information. | 21 | 28 | 0 | 0 | 0 | 0 | 0 | 9 | 0.75 | 0.795 |
| 12 | The patient cannot stay focused on an activity for an extended time (e.g. when following a conversation or performing a task). | 39 | 52 | 0 | 0 | 0 | 0 | 0 | 9 | 0.32 | 0.831 |
|  | **Perception** |  |  |  |  |  |  |  |  |  |  |
| 13 | The patient does not recognise objects, or mistakes them for something else. | 70 | 93.3 | 2 | 2.7 | 0 | 0 | 0 | 6 | 0.45 | 0.699 |
| 14 | The patient does not recognise familiar faces. | 63 | 84 | 3 | 4 | 0 | 0 | 0 | 6 | 0.12 | 0.726* |
| 15 | The patient does not recognise letters, symbols and/or numbers. | 63 | 84 | 6 | 8 | 0 | 0 | 0 | 4 | 0.41 | 0.681 |
| 16 | The patient does not recognise sounds or does not respond adequately to sounds (e.g. sound of alarm clock). | 70 | 93.3 | 4 | 5.3 | 0 | 0 | 0 | 1 | 0.00 | 0.722* |
| 17 | The patient gets lost in a familiar environment. | 57 | 76 | 5 | 6.7 | 0 | 0 | 0 | 9 | 0.83 | 0.504 |
| 18 | The patient gets lost in an unfamiliar environment (e.g. supermarket or other department of the care facility). | 32 | 42.7 | 26 | 34.7 | 0 | 0 | 0 | 9 | 0.85 | 0.507 |
| 19 | The patient cannot estimate the distance to or position of an object in relation to him/herself and therefore misses the object when reaching for it. | 62 | 82.7 | 1 | 1.3 | 0 | 0 | 0 | 6 | 0.50 | 0.658 |
|  | **Orientation & memory** |  |  |  |  |  |  |  |  |  |  |
| 20 | The patient does not know where he/she is. | 63 | 84 | 1 | 1.3 | 0 | 0 | 0 | 2 | 0.48 | 0.802 |
| 21 | The patient does not know whether it is morning, afternoon or evening. | 62 | 82.7 | 1 | 1.3 | 0 | 0 | 0 | 6 | 0.48 | 0.795 |
| 22 | The patient does not know what day it is. | 54 | 72 | 3 | 3.4 | 0 | 0 | 0 | 4 | 0.41 | 0.8 |
| 23 | The patient does not know his/her own personal data (address, date of birth, etc.). | 68 | 90.7 | 5 | 6.7 | 0 | 0 | 0 | 4 | 0.09 | 0.817* |
| 24 | Patient does not remember autobiographical information (e.g. personal events in the past). | 63 | 84 | 3 | 4 | 0 | 0 | 0 | 2 | 0.39 | 0.808 |
| 25 | The patient does not show up to scheduled appointments. | 41 | 54.7 | 7 | 9.3 | 1 | 1.3 | 0 | 9 | 0.36 | 0.804 |
| 26 | The patient cannot recall recent information that has to be applied immediately (e.g. instructions on how to perform a task). | 42 | 56 | 0 | 0 | 1 | 1.3 | 0 | 9 | 0.71 | 0.763 |
| 27 | The patient is not able to learn new tasks (habit formation) (e.g. operating a new coffee machine). | 37 | 49.3 | 10 | 13.3 | 0 | 0 | 0 | 9 | 0.61 | 0.778 |
| 28 | The patient is not able to continue an activity after an interruption (e.g. when asked a question), because the patient does not remember what he/she was doing. | 39 | 52 | 2 | 2.7 | 0 | 0 | 0 | 9 | 0.63 | 0.777 |
| 29 | The patient has difficulty remembering recent events, not even when asked to recall a specific event (e.g. remember what we had for dinner yesterday?). | 44 | 58.7 | 1 | 1.3 | 0 | 0 | 0 | 9 | 0.72 | 0.763 |
| 30 | The patient doesn’t spontaneously remember previously retained information but does recognize it when it’s mentioned (e.g. remember we had pasta for dinner yesterday?) | 41 | 54.7 | 5 | 6.7 | 0 | 0 | 0 | 9 | 0.45 | 0.799 |
|  | **Actions** |  |  |  |  |  |  |  |  |  |  |
| 31 | The patient performs the parts of an action in an incorrect order (i.e. apraxia) (e.g. when getting dressed). | 59 | 78.7 | 4 | 5.3 | 0 | 0 | 0 | 9 | 0.663 | 0.075 |
| 32 | The patient uses objects in an incorrect, clumsy or unsafe way. | 63 | 84 | 1 | 1.3 | 0 | 0 | 0 | 9 | 0.691 | 0.013 |
| 33 | The patient uses the wrong object to perform an activity (e.g. uses a comb to clean his/her teeth). | 71 | 94.7 | 2 | 2.7 | 0 | 0 | 0 | 2 | 0.057 | 0.801* |
|  | **Language and communication** |  |  |  |  |  |  |  |  |  |  |
| 34 | The patient does not respond adequately to spoken language (e.g. does not understand the meaning). | 61 | 81.3 | 0 | 0 | 0 | 0 | 0 | 9 | 0.738 | 0.825 |
| 35 | The patient does not respond to spoken information consisting of several sentences (e.g. a route description and/or instructions). | 56 | 74.7 | 3 | 4 | 0 | 0 | 0 | 9 | 0.765 | 0.810 |
| 36 | The patient does not understand figurative language and/or metaphors and humour (e.g. ‘my hands are tied'/'still waters run deep'). | 58 | 77.3 | 8 | 10.7 | 0 | 0 | 0 | 6 | 0.368 | 0.831 |
| 37 | The patient cannot come up with correct words or names. | 46 | 61.3 | 0 | 0 | 0 | 0 | 0 | 9 | 0.586 | 0.817 |
| 38 | The patient says other words than what he/she means (paraphasia). | 60 | 80 | 0 | 0 | 0 | 0 | 0 | 9 | 0.583 | 0.817 |
| 39 | The patient cannot verbally express what he/she means (exactly). | 43 | 57.3 | 0 | 0 | 0 | 0 | 0 | 9 | 0.584 | 0.817 |
| 40 | The patient cannot formulate grammatically correct sentences. | 60 | 80 | 2 | 2.7 | 0 | 0 | 0 | 9 | 0.517 | 0.820 |
| 41 | The patient cannot understand single written words. | 66 | 88 | 5 | 6.7 | 0 | 0 | 0 | 6 | 0.405 | 0.828 |
| 42 | The patient cannot understand written sentences and/or texts. | 61 | 81.3 | 6 | 8 | 0 | 0 | 0 | 9 | 0.556 | 0.821 |
| 43 | The patient cannot write single words. | 51 | 68 | 16 | 21.3 | 0 | 0 | 0 | 9 | 0.497 | 0.825 |
| 44 | The patient cannot write sentences and/or texts. | 43 | 57.3 | 22 | 29.3 | 0 | 0 | 0 | 9 | 0.621 | 0.814 |
| 45 | When telling a story, the patient cannot distinguish between what is important and what is less important (is not to the point). | 40 | 53.3 | 2 | 2.7 | 0 | 0 | 0 | 9 | 0.516 | 0.820 |
| 46 | The patient finds it hard to stay on topic and drifts off topic in conversation (i.e. tangential speech). | 45 | 60 | 1 | 1.3 | 0 | 0 | 0 | 9 | 0.437 | 0.826 |
| 47 | The patient does not make eye contact in a conversation. | 60 | 80 | 0 | 0 | 0 | 0 | 0 | 9 | -0.008 | 0.850* |
| 48 | The patient interrupts others in a conversation. | 63 | 84 | 0 | 0 | 0 | 0 | 0 | 9 | 0.306 | 0.832 |
| 49 | The patient does not start a conversation. | 68 | 90.7 | 0 | 0 | 0 | 0 | 0 | 1 | 0.181 | 0.836* |
|  | **Task behaviour** |  |  |  |  |  |  |  |  |  |  |
| 50 | The patient cannot really determine what needs to be done to achieve his/her goal when performing a new activity. | 23 | 30.7 | 5 | 6.7 | 1 | 1.3 | 0 | 9 | 0.56 | 0.937 |
| 51 | The patient sets unrealistic goals (e.g. being able to work full-time immediately after being away for a long time). | 39 | 52 | 2 | 2.7 | 0 | 0 | 0 | 9 | 0.77 | 0.939 |
| 52 | The patient only sets short-term goals (activity), no medium-term (day or week planning) and/or long-term (months, years) goals. | 38 | 50.7 | 10 | 13.3 | 0 | 0 | 0 | 9 | 0.70 | 0.943 |
| 53 | The patient cannot think of solutions to a problem. | 27 | 36 | 2 | 2.7 | 0 | 0 | 0 | 9 | 0.87 | 0.942 |
| 54 | The patient does not prioritise his/her activities. | 38 | 50.7 | 5 | 6.7 | 0 | 0 | 0 | 9 | 0.78 | 0.936 |
| 55 | The patient does not apply a step-by-step or efficient approach during an everyday task (e.g. when looking for an object). | 33 | 44 | 2 | 2.7 | 0 | 0 | 0 | 9 | 0.75 | 0.936 |
| 56 | The patient is not able to execute a scheduled plan. | 39 | 52 | 2 | 2.7 | 1 | 1.3 | 0 | 9 | 0.88 | 0.935 |
| 57 | The patient does not anticipate events or activities (e.g. does not put on a coat when going outside because he/she didn’t anticipate the weather conditions). | 48 | 64 | 9 | 12 | 0 | 0 | 0 | 9 | 0.73 | 0.939 |
| 58 | The patient does not perform everyday activities on his/her own initiative but performs these actions readily with prompting (e.g. daily routines such as walking the dog or washing the dishes after dinner). | 45 | 60 | 8 | 10.7 | 0 | 0 | 0 | 9 | 0.86 | 0.941 |
| 59 | The patient does not start non-routine activities on his/her own initiative but performs these actions readily with prompting (e.g. making an appointment at the hairdresser's). | 34 | 45.3 | 15 | 20 | 0 | 0 | 0 | 9 | 0.79 | 0.939 |
| 60 | The patient starts an activity without thinking or planning. | 43 | 57.3 | 1 | 1.3 | 0 | 0 | 0 | 9 | 0.68 | 0.941 |
| 61 | The patient cannot control him/herself (i.e. stop inadequate behaviour on own initiative) (e.g. eats food when in sight of it or makes inappropriate remarks). | 58 | 77.3 | 2 | 2.7 | 0 | 0 | 0 | 9 | 0.54 | 0.942 |
| 62 | The patient is upset when plans change. | 38 | 50.7 | 3 | 4 | 0 | 0 | 0 | 6 | 0.43 | 0.946* |
| 63 | The patient cannot switch to new behaviour/another procedure if the situation requires this. | 31 | 41.3 | 2 | 2.7 | 2 | 2.6 | 0 | 9 | 0.74 | 0.941 |
| 64 | The patient does not monitor what he/she is doing and fails to make corrections if necessary. | 32 | 42.7 | 2 | 2.7 | 0 | 0 | 0 | 9 | 0.89 | 0.941 |
| 65 | The patient fills the gaps in his/her memory or perception with incorrect information and is not able to judge the representation as false (confabulates). | 49 | 65.3 | 6 | 8 | 1 | 1.3 | 0 | 9 | 0.39 | 0.943 |

**Supplemental Figure A.** Histograms of the score distributions for unscaled scores on each CDL subscale.

*Note:* CDL = cognition in daily life scale.

**Supplemental Figure B.** Scatter plots of relationships between Cognition in Daily Life subscales and the Cognition subscale of the Utrecht Scale for the Evaluation of Rehabilitation.

*Note:* CDL = cognition in daily life scale; USER = Utrecht Scale for the Evaluation of Rehabilitation.

**Supplemental Figure B.** Scatter plots of relationships between Cognition in Daily Life subscales and the Montreal Cognitive Assessment.

*Note:* CDL = cognition in daily life scale; MoCA = Montreal Cognitive Assessment.
